# Oncofertility as an Essential Part of Comprehensive Cancer Treatment in Patients of Reproductive Age, Adolescents and Children

**DOI:** 10.3390/cancers16101858

**Published:** 2024-05-13

**Authors:** Dominika Łubik-Lejawka, Iwona Gabriel, Adrianna Marzec, Anita Olejek

**Affiliations:** Department of Gynaecology, Obstetrics and Oncological Gynaecology in Bytom, Medical University of Silesia, 40-055 Katowice, Poland; dominikalubik@gmail.com (D.Ł.-L.); iwona.gabriel@sum.edu.pl (I.G.); adrianna.jurkiewicz@interia.pl (A.M.)

**Keywords:** oncofertility, fertility preservation, reproductive potential, gonadotoxicity, cryopreservation, ovarian suppression, oophoropexy

## Abstract

**Simple Summary:**

The number of oncological patients of prepubertal and reproductive age is increasing steeply worldwide. Not only have medical innovations and scientific advances over the last few decades enabled more and more patients to survive, but also, they have revolutionised the processes of preserving and restoring fertility. However, not every fertility preservation method is optimal and appropriate after specific oncological treatment, and some of them remain experimental. Therefore, there is a constant need to develop and improve the field of oncofertility. Additionally, governments should facilitate access to fertility preservation programmes for oncological patients and should look after their psychological needs as well.

**Abstract:**

The number of children, adolescents and young adults diagnosed with cancer has been rising recently. Various oncological treatments have a detrimental effect on female fertility, and childbearing becomes a major issue during surveillance after recovery. This review discusses the impact of oncological treatments on the ovarian reserve with a thorough explanation of oncologic treatments’ effects and modes of oncofertility procedures. The aim of this review is to help clinicians in making an informed decision about post-treatment fertility in their patients. Ultimately, it may lead to improved overall long-term outcomes among young populations suffering from cancer.

## 1. Introduction

Considering GLOBOCAN global statistics, the number of new cancer cases in children and among adolescents and young adults (AYA, 15–39 years old) reached almost 1.5 million cases in 2020 [1]. The types of neoplasms that are most commonly diagnosed among 0–14-year-old children are leukaemia, Bing–Neel syndrome (BNS) and non-Hodgkin lymphoma (NHL) (32.5%, 11.8% and 9.2%, respectively). Adolescents and young adults are most frequently diagnosed with breast, thyroid and cervical cancers (19.8%, 12.1% and 9%, respectively) [1,2]. In the United States alone, the number of paediatric and AYA survivors was nearly 800,000 cases in 2022, with the female sex being predominant [3]. The 5-year survival rates are approximately 85% in both the paediatric and AYA group [4,5]. According to the EUROCARE data, in European countries, the heterogeneity in survival statistics between individual countries as well as regions—Eastern and Western Europe—is notable. The heterogeneity is partly caused by differences in the incidence of particular neoplasms, but the main reasons are thought to be discrepancies in the diagnostic and therapeutic methods available in different regions [6]. Despite regional differences, a significant increase in 5-year survival rates has been observed over the past few decades, from 30% in the 1960s to more than 80% today [7]. Survival rates are projected to continue their upward trend over the next decade, with a global increase of just over 20% [8]. Improving the survival of childhood- and reproductive-age oncology patients brings a real need to effectively safeguard their fertility, which is threatened with aggressive oncology therapies.

The first oncofertility association was established in 2007 in the United States and named the Oncofertility Consortium [9]. Initially, it brought together national centres, whereas today, it unites 45 centres around the world. It aims to support research and accelerate the development of new therapeutic and fertility-preserving techniques for patients, whose fertility may be compromised due to cancer, non-cancer diseases and iatrogenic factors [9,10,11].

Factors that compromise female reproductive potential may lead to subfertility or infertility. Infertility is a condition diagnosed approximately in one in eight women of reproductive age. It is defined as a lack of ability to achieve pregnancy after 6 to 12 months of regular sexual intercourse without using any methods of contraception. Ovulatory dysfunction, tubal disorders, uterine factors, endometriosis, male factor and primary ovarian insufficiency (POI) are among the major identifiable causes and constitute about 85% [12]. Environmental and lifestyle-related factors may also contribute to the deterioration of female fertility. They involve inadequate diet, obesity, stress, circadian clock disturbance, smoking, alcohol consumption and a range of various chemicals and air pollutants [13]. In fifteen percent of infertile couples, the cause of infertility is unknown [14]. The ESHRE defines POI as the absence of menstruation lasting ≥4 months in women under 40 years old, with two FSH measurements exceeding 25 IU/l. FSH measurements should be performed at least 1 month apart [15]. Many causes of POI remain unknown. The pivotal ones that have been identified involve genetic disorders that account for up to 20–25% of cases and are correlated to single gene mutations and chromosomal abnormalities [16,17]. Another 20% of POI cases result from immune system disorders, which most commonly are autoimmune thyroid conditions [18,19]. Iatrogenic factors leading to POI comprise ovarian surgery, chemotherapy and radiotherapy. Multi-faceted mechanisms leading to POI are identified within this group. Having an impact directly on ovarian follicles, they reduce the non-renewable pool of primordial follicles and deteriorate oocyte quality through the damage of intracellular DNA. Additionally, they affect ovarian stroma leading to its fibrosis and causing damage to the vascular endothelial cells [11,20,21].

This review summarises the influence of cancer treatment strategies on women’s fertility. It also discusses methods of fertility preservation, ranging from fertility-sparing surgery for gynaecologic cancers, the cryopreservation of oocytes and embryos, the cryopreservation of ovarian tissue, in vitro maturation (IVM), ovarian transposition (oophorectomy) and ovarian suppression to experimental methods.

## 2. Reproductive Potential Assessment

The ovarian reserve is a pool of primordial follicles determined at birth and estimated at around 1,000,000–2,000,000. It undergoes progressive atresia so that around 400,000 follicles remain at puberty. The primordial follicles are arrested in the prophase stage of the first meiotic division, until they are activated into growing follicles from puberty onwards. The process of activation occurs periodically until menopause, when the pool of primordial follicles is exhausted. Female age affects not only the quantitative component of the ovarian reserve but also the qualitative one, which is related to the risk of genetic abnormalities in the embryo. The qualitative component more accurately than the quantitative one correlates with the woman’s age and is coherent between different populations, whereas the quantitative component can vary significantly between women of the same age [22]. The qualitative component is influenced by disturbances including chromatid cohesion, meiotic recombination, division spindle checkpoint (SAC) function, DNA repair processes, telomerase activity, reactive oxygen species production and mitochondrial DNA (mtDNA) function whose frequency increases with age [23]. Reduced ovarian reserve is affected not only by age but also by genetic factors; autoimmune factors; smoking; iatrogenic factors including ovarian surgery, chemotherapy and radiotherapy; changes in follicular fluid content; the composition of the vaginal microflora and environmental factors [24]. Basic tests assessing the ovarian reserve include measurements of anti-Mullerian hormone (AMH), follicle-stimulating hormone (FSH), oestradiol (E2) levels and antral follicle count (AFC).

### 2.1. AMH

AMH is a dimeric glycoprotein that belongs to the transforming growth factor β (TGFβ) family. Its gene is located on the short arm of chromosome 19 and consists of five exons. AMH is synthesised in a form of a 560 amino acids homodimer precursor, which after dimerisation, is cleaved into the noncovalent complex of N- and C-terminal domains. Not only does AMH bind to receptors type 1 and SMAD like other members of the TGFβ family, but it also has a dedicated ligand-specific receptor—AMH receptor type 2 (AMHR2) [25]. AMH binds to AMHR2 by its biologically active C-terminal domain. The gene for AMHR2 is located on the long arm of chromosome 12 and consists of 11 exons [26]. It has a fairly stable concentration throughout the whole menstrual cycle apart from a slight decrease in its level at the end of the follicular phase; hence, its value can be measured irrespective of the day of the cycle. AMH is secreted by the granulosa cells of growing follicles, mainly preantral and early antral follicles, and thus can be used to evaluate the functional ovarian reserve [22,27,28]. Despite being a good predictor of the number of follicles that can potentially ovulate, AMH does not inform about their quality or real chance of becoming pregnant or pregnancy progression [22]. In practice, AMH measurements are most commonly used in the prediction of menopausal age and in the diagnostics and treatment of infertility in order to tailor the stimulation protocols during IVF procedures to individual patients’ needs and estimate the risk of poor response or ovarian hyperstimulation syndrome (OHSS) [28]. In gynaecologic oncology, AMH is used to assess the extent of damage to the ovarian reserve as a result of gonadotoxic treatment. A number of studies have demonstrated that AMH levels before the commencement of therapy are a sensitive predictor of reproductive potential after treatment [29,30,31,32]. Although no correlation has been shown between AMH levels before oncological treatment and its fluctuations in response to treatment, patients having higher levels of the hormone before therapy maintained higher levels after therapy [33]. Apart from the pre-treatment AMH level, the post-therapy AMH value is also influenced by the woman’s age at the beginning of treatment and the type of chemotherapeutics used [34,35].

### 2.2. AFC

AFC consists of an ultrasound measurement of antral follicles fulfilling particular criteria. Follicles having a diameter between 2 and 10 mm from both ovaries are added up. The test should be performed in the early follicular phase, between days 2 and 4 of the menstrual cycle, and it should detect >5 antral follicles. AFC is characterised by low variability between menstrual cycles. The majority of scientific papers have described high consistency between AMH and AFC measurements in determining female reproductive potential [35,36]; hence, AFC is considered a reliable equivalent to AMH measurement. Although chemotherapy results in a decrease in AFC, 40% of its pre-treatment value is restored 1 year after therapy. Quicker AFC recovery and to a greater degree of 56% is possible by suppressing the ovaries during chemotherapy by adding a GnRH agonist [37].

### 2.3. Inhibin B

Inhibin B, like AMH, is a glycoprotein belonging to the TGFβ family. It is secreted by the granulosa and thecal cells of preantral follicles. It should be measured in the early follicular phase—on day 2 of the menstrual cycle. Inhibin B shows a negative feedback loop with FSH. A decrease in its concentration leads to an increase in FSH levels, which consequently shortens the follicular phase and the length of the menstrual cycle [38]. Cancer survivors who have been exposed to gonadotoxic therapy have lower levels of inhibin B compared to healthy women of the same age [39].

### 2.4. FSH and E2

FSH is a pituitary hormone which influences the synthesis of oestradiol and progesterone in the ovary. It is measured in the early follicular phase, between days 2 and 4 of the menstrual cycle. FSH shows a high intra- and inter-cyclic variability; hence, a single measurement does not reliably determine ovarian reserve [38]. Higher FSH and lower E2 measurements after chemotherapy are consistent with decreased post-treatment levels of inhibin B [39].

All discussed above fertility assessment markers are similarly affected by radiotherapy (Table 1) [40].

## 3. Impact of Therapeutic Methods on Fertility

### 3.1. Chemotherapy

There are five classes of chemotherapeutic drugs differing in the mode of action and effect on fertility. Alkylating agents involve platinum coordination complexes, nitrogen mustards, ethyleneimines, alkyl sulfonates, nitrosoureas and triazenes. Antimetabolites include folic acid antagonists, pyrimidine antimetabolites and purine antimetabolites. Vincristine and vinblastine belong to the vinca alkaloid group. Antitumour antibiotics consist of anthracyclines, bleomycin, dactinomycin and mitomycin. Taxanes include paclitaxel and docetaxel.

**Figure 1:**
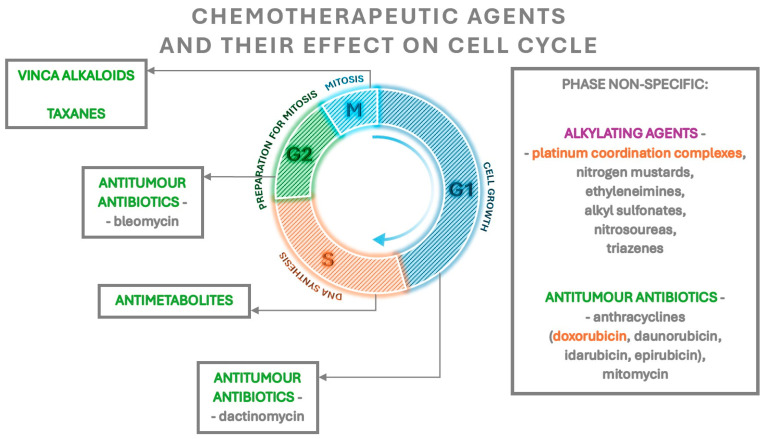
The effect of chemotherapeutic agents on the cell cycle. The highest gonadotoxic risk agents are marked in purple, the intermediate risk agents marked in orange and agents with a lower risk marked in green.

Alkylating drugs, except for platinum coordination complexes whose risk is defined as intermediate, pose the highest risk of fertility loss. The other groups are characterised by a lower risk, with the exception of doxorubicin, which belongs to anthracyclines and has an intermediate risk (Figure 1) [41,42]. Not only does the gonadotoxic effect of cytotoxic drugs depend on their type but also on the dose, the route of administration, the type of neoplasm and the ovarian reserve at the beginning of treatment [11,41].

Each group of chemotherapeutics has a slightly different effect. Alkylating drugs are cell cycle non-specific—they act on cells independently of the phase of the cycle. Their active metabolites alkylate the nitrogen atoms of the nucleotide bases. This leads to forming cross-links in the DNA and damaging the DNA strand structure by the formation of breaks in the DNA strand. Subsequently, the DNA replication process is disrupted. Some alkylating agents also block enzymes that mediate DNA repair processes [43]. These drugs significantly reduce the number of ovarian follicles compared to other groups of chemotherapeutics. Not only do they induce the atresia of primordial and growing follicles, but they also enhance the activation of the former [44,45]. The effect, described as “burnout”, is a reduction in the secretion of inhibitory factors by atretic growing follicles. This in turn leads to an increase in the phosphorylation of proteins involved in the PI3K/PTEN/Akt and mTOR/PTEN/Akt pathways, such as Akt, PI3K, mTOR and rpS6, that enhance the activation of primordial follicles and consumption of their pool [45,46]. The atresia of primordial follicles occurs by the apoptosis of oocytes, whereas the apoptosis of the granulosa cells of these follicles is not observed (Table 2) [47,48].

Doxorubicin, which belongs to the group of anthracyclines, is characterised by intermediate risk. It exhibits phase-non-specific activity—its greatest activity is manifested against cells in S, G2 and M phases [43]. The molecular mechanism of its action in the cell is not fully elucidated, but studies report that its molecules penetrate between DNA base pairs [49]. This leads to changes in the distance between bases, followed by changes in the helix torsion angle and the disruption of its structure and subsequent disturbances in replication and transcription processes, which are primarily attributed to the gonadotoxicity of doxorubicin. In addition, the inhibition of topoisomerase II activity, disruption of mitochondrial function and exacerbation of cellular oxidative stress have been described [49,50]. Doxorubicin, in contrast to alkylating drugs, leads to the atresia of primordial and growing follicles through damaging granulosa cells within them, rather than oocytes [51,52]. As with alkylating drugs, a mechanism for decreasing the pool of primordial follicles through their increased activation is also described. Furthermore, the atresia and increased activation of primordial follicles show an age-dependent effect—the number of follicles lost increases with the age of the treated patient [53]. Apart from reducing the number of primordial follicles, alkylating drugs and doxorubicin also affect their quality by increasing the number of morphologically abnormal follicles (Table 2). This is manifested by the presence of oocytes with little eosinophilic cytoplasm and/or with condensed nuclear chromatin, as well as the presence of abnormally shaped granulosa cells and/or with condensed chromatin [51,52]. In addition, these drugs enhance the apoptosis of ovarian stroma cells and reduce their ability to divide [51], causing local inflammation, focal ovarian cortex fibrosis and the apoptosis of endothelial cells leading to impaired endothelial function and reduced vascular blood flow, followed by the further impaired development and atresia of ovarian follicles [45,50,54].

The action of antimetabolites is phase-specific as they act in the S phase of the cell cycle. These drugs cause a competitive blockage of the enzymatic reactions responsible for tumour growth [43]. Regarding their effects on the ovaries, they primarily cause growth inhibition and atresia in the pool of growing preantral and antral follicles, without reducing the number of primordial follicles (Table 2) [55]. Rapidly dividing granulosa cells in preantral and antral follicles have a high metabolic demand and hence are more sensitive to the effects of antimetabolites, which explains their selective atresia [27,44].

Taxanes are a group of drugs that inhibit the function of the division spindle by binding to tubulin and stabilising microtubules. In addition, these drugs induce the apoptosis of cells by inducing their return to the G phase of the cell cycle [27]. Much controversy revolved around the specific gonadotoxicity of taxanes due to the fact that they were usually used in combination or sequential therapies [56]. Initial reports did not indicate harmful effects of their use, but later studies confirmed their gonadotoxicity involving the induction of ovarian growing follicle atresia by the inhibition of the function of division spindles during meiotic division I and II, leading to endocrine disruption, amenorrhoea and reduced ovarian reserve (Table 2) [27,56,57,58,59].

Vinca alkaloids (vincristine, vinblastine) are phase-specific, acting mainly in the M phase of the cell cycle, and their mechanism is similar to that of taxanes—by binding to tubulin and inhibiting microtubule polymerisation, they lead to the inhibition of mitotic spindle function [43]. To date, there is no indication that they cause long-term gonadotoxicity; studies in mice have shown that these drugs result in the atresia of growing follicles but have no effect on the pool of primordial follicles (Table 2) [60].

### 3.2. Radiotherapy

Radiotherapy, by adversely affecting the ovaries, increases the risk of POI, and by causing changes in the uterus, it not only results in reduced fertility but, if pregnancy is achieved, increases the risk of pregnancy complications and adverse neonatal outcomes. The risk of ovarian follicle damage varies according to the radiation dose, the type of field irradiated, the dose fractionation scheme and the phase of follicle development, of which the periovulatory phase is the most sensitive [61]. Oocytes are impaired at doses of 1–5 Gy [61,62]; in a study by Wallace et al., the median lethal dose (LD50) was estimated to be <2 Gy [63]. The dose leading to infertility immediately after treatment is considered to be about 19 Gy in children and about 15 Gy in adults, a dose that decreases with age [64,65]. Irradiation also results in the fibrosis of the ovarian stroma through stromal vascular injury [66].

Furthermore, radiation induces changes in the myometrium causing its thinning and fibrosis and endothelial vascular damage in the uterus [67]. These changes manifest themselves as a reduction in the size of the organ, the degree of which correlates with the age at which irradiation was applied. The younger the age, the greater the reduction in the organ size and the lesser the response to treatment with hormone replacement therapy [68]. Changes in the uterus result in a lower rate of live births and a higher rate of miscarriages, preterm births and low neonatal birth weight if a pregnancy is achieved, despite fertility-preserving procedures [68,69]. Lower live birth rates are a consequence of the lesions formed in the irradiated uterus and also the compromised vascularisation of the ovarian tissue graft resulting from the fibrosis of the pelvic tissues [62]. Irreversible uterine damage, at which pregnancy is contraindicated, is observed at radiation doses of 25 Gy applied in childhood and 45 Gy applied in adulthood [62,70].

Total body irradiation (TBI) usually applied at a dose of 12 Gy, as a procedure preparing patients to receive a bone marrow transplant, also carries a risk of reduced fertility and negative prenatal and neonatal outcomes [62,70].

Radiotherapy to the central nervous system, on the other hand, leads to hormonal changes that may affect fertility. Brain irradiation causes damage to pituitary cells and leads to hypopituitarism. Doses of 30–40 Gy result in secondary hypogonadism in 80% of treated patients. Another parameter evaluating the function of the hypothalamic area after radiotherapy is the prolactin level. It has been reported that hyperprolactinaemia after brain irradiation can affect up to 50% of patients [71,72].

### 3.3. Surgical Treatment

Women’s reproductive potential may be directly affected by undergoing gynaecological surgery, both oncological and non-oncological. Adnexal surgery can reduce the pool of ovarian follicles or deprive patients of it completely. After undergoing a hysterectomy, the only chance of having offspring is surrogacy. Sparing surgeries, requiring advanced qualifications, remain a chance to preserve fertility. Cervical cancer is the most common of all gynaecological cancers, ranking fourth among all female cancers [1]. Sparing treatment can be applied to squamous cell carcinoma or adenocarcinoma developed on the basis of HPV infection, with a maximum tumour size of 2 cm, without lymph node metastases present. An important stage in the qualification for sparing treatment is the evaluation of the sentinel node. Patients found to have rare histological types should not be eligible for sparing treatment. Fertility-sparing surgery is an alternative to radical hysterectomy and its aim, apart from excising the tumour with an adequate margin of healthy tissue, is to preserve the upper part of the cervix. Patients in stage T1a1 and T1a2 with negative lymph nodes, regardless of the presence of lymphovascular space invasion (LVSI), are offered conisation and simple trachelectomy. Patients at stage T1b1 with negative lymph nodes and without the infiltration of the lymphovascular spaces may also undergo conisation and simple trachelectomy, or a radical trachelectomy may be considered. Patients at stage T1b1 with lymphovascular space invasion should be qualified for radical trachelectomy—type B. During trachelectomy, a transabdominal isthmic circular suture should be placed intraoperatively [73].

Endometrial cancer is the sixth most commonly diagnosed cancer among women worldwide, and its incidence is increasing [1]. Although it is mainly diagnosed in postmenopausal women, 4% of patients are women younger than 40 years [74]. The standard surgical procedure is a simple removal of the uterus with the adnexa. Fertility-sparing treatment may be given to patients in the early stages of the disease, without metastases—those diagnosed with endometrial intraepithelial metaplasia (EIN) or G1 endometrioid carcinoma not infiltrating the myometrium. Sparing treatment consists of hormonal therapy with medroxyprogesterone acetate at a dose of 400–600 mg/d, megestrol acetate at a dose of 160–320 mg/d or an IUD containing levonorgestrel at a dose of 52 mg in combination with or without oral progestogens. At present, the combination of hysteroscopic resection with subsequent hormone therapy also appears reasonable and is characterised by a complete response rate in 95.3% of cases and a recurrence rate of 14.1% [75]. Complete responses after hormone therapy alone are achieved in almost 80% [76]. Histopathological and imaging verification is required again 6 months post treatment. If there is a complete response to hormonal treatment, the patient may try to become pregnant. If obesity or insulin resistance is present, the time needed to achieve a response may be longer, in which case it is justified to continue therapy for up to 12 months [77,78].

Twelve percent of ovarian cancer cases affect women of reproductive age [79]. Fertility-sparing treatment consists of preserving the unoccupied ovary and uterus. It is possible in patients with low-grade IA and IC1 tumours of serous, endometrial or mucinous type with an expanding growth type [80]. In case of a lesion limited to one ovary and with a normal appearance of the second ovary, a biopsy of the second ovary is not recommended, as this may result in a reduction in ovarian reserve or the formation of adhesions and thus reduced fertility. The risk of cancer in the contralateral ovary has been estimated to be only 3%. For borderline tumours, unilateral salpingo-oophorectomy or unilateral ovarian cystectomy is performed. The first method is preferred as it has a risk of recurrence up to 8 per cent, compared to up to 30 per cent after cystectomy [81]. Sparing treatment can also be considered in stage IB and usually consists of the enucleation of the tumour from the ovary where it is well delineated [82]. The qualification of patients for fertility-sparing treatment should be very meticulous, and in addition to the stage of the tumour, it should include an assessment of reproductive potential and risk factors that may affect the success of becoming pregnant and the delivery of a pregnancy.

## 4. Fertility Preservation Methods

### 4.1. Cryopreservation of Oocytes and Embryos

Currently, the first-line fertility preservation method in women is the cryopreservation of oocytes or embryos. It is effective and widely available. Typically, these must be preceded by a controlled ovarian hyperstimulation (COH) procedure. There is some controversy over the use of COH which is related to the limited time prior to the commencement of oncological treatment and the oestrogen-dependent behaviour of some cancers. According to the ESHRE recommendations, the preferred COH protocol is with GnRH antagonists which require a shorter stimulation time and have a lower risk of complications, i.e., OHSS. The stimulation of the ovulation process can also be shortened by using a random-start protocol and a double stimulation protocol, called Shanghai protocol, which involves double stimulation in the same cycle, in the follicular and luteal phases. Longer protocols may be considered if more time is available before the initiation of cancer treatment. For oestrogen-dependent cancers, concomitant anti-oestrogen therapy in the form of letrozole should be considered [83]. Anti-oestrogen therapy enables using lower doses of gonadotropins and thus lower oestrogen concentrations during stimulation. Having obtained a greater number of oocytes than in the natural cycle through controlled ovarian hyperstimulation, they undergo cryopreservation (freezing in liquid nitrogen), either directly after stimulation or following fusion with sperm and forming embryos. The most commonly used technique for this is vitrification, which is high-speed freezing. It has replaced the previously applied slow freezing, the drawback of which was the precipitation of ice crystals in the frozen preparation, posing a greater risk of structural damage and impaired cell function. Vitrification is a less time-consuming method and does not require expensive equipment. Furthermore, it has been proven in studies that embryos undergoing vitrification have a higher survival rate after thawing than the ones undergoing slow freezing as the vitrification method has less impact on their structure and leads to better clinical outcomes [84,85]. The age of patients affects the number of stimulation cycles required, the number of oocytes obtained, the doses of gonadotropins used for hyperstimulation and the percentage of live births; results in all the aforementioned categories worsen with increasing age [86,87]. Disregarding the age of patients, implantation and live birth rates after oocyte cryopreservation are lower in patients who have undergone oncological treatment compared to non-oncology patients undergoing this procedure, which may indicate the influence of cancer per se, but this has not been proven in studies [86,87,88,89]. The cryopreservation of oocytes or embryos does not carry an increased risk of birth defects and chromosomal aberrations in the offspring [83,90]. In a meta-analysis by Fraison et al., it was summarised that the live birth rate following IVF with cryopreserved oocytes was 32%, while the percentage of miscarriages ranged from 10% to 15%. In the same publication, the live birth rate after the cryopreservation of embryos was estimated to be 41%, and the proportion of women with miscarriages was 22% [91].

### 4.2. Cryopreservation of Ovarian Tissue

Ovarian tissue cryopreservation (OTC) with subsequent ovarian tissue transplantation (OTT) has been recognised by scientific societies as a therapeutic method used to preserve fertility in oncological patients. This procedure is dedicated to prepubertal patients and those in whom urgent oncological treatment makes it impossible to undergo controlled ovarian hyperstimulation. Depending on the size of the ovary and ovarian reserve, ovarian tissue can be retrieved by an ovarian cortex biopsy (OCB) (large ovary size and high ovarian reserve) or unilateral removal of the entire ovary (small ovary size and low ovarian reserve) [92]. A biopsy of the ovarian cortex remains a less invasive method, and the remaining ovaries can be used as a site for retransplantation. The procedure can be performed regardless of the menstrual cycle phase. The retrieved tissue is divided into smaller 1–2 mm fragments before freezing, using the ‘slow-freezing’ method [83]. Following the completion of the oncological therapy, the frozen stored ovarian tissue undergoes thawing and transplantation. The fragments are transplanted either orthotopically—into the pelvis, into an anatomically compatible site (left ovaries, broad ligament, peritoneal pocket by the ovary)—or heterotopically—into non-anatomical sites (subcutaneous tissue of the forearm, abdominal shell). In addition to restoring procreative function, the transplantation of the ovarian tissue restores hormonal function, regardless of location (ortho- and heterotopic). From studies, it appears that orthotopic transplantation allows for the obtaining of higher quality gametes and embryos and hence should be preferred in women wishing to achieve pregnancy. In contrast, heterotopic transplantation, as a less invasive approach, should be considered mainly in women wishing to solely achieve a return of endocrine function [93]. Moreover, orthotopic transplantation may be followed by natural conception, thereby being the only method that avoids other medical procedures. However, conception rates after natural conception and IVF are similar and constitute 40% and 36%, respectively. The live birth rate in women conceiving naturally stands at 30%, while the miscarriage rate is around 10%. In comparison, the live birth rate following IVF is lower and constitutes 21%, while the miscarriage rate is higher standing at 18% [62]. The available data indicate a significant influence of age and ovarian reserve on the effectiveness of fertility restoration after OTC/OTT; hence, the procedure should not be considered in patients over 36 years of age and with a low ovarian reserve of AMH < 0.5 ng/mL or AFC < 5 [83]. Potential problems of the procedure include risks associated with surgery, the implantation of tumour cells present in the transplanted tissue and bacterial infections. Most often, OTC/OTT involves undergoing two surgical procedures, and although they are usually minimally invasive laparoscopies, there is a small risk of occurrence of the typical complications associated with this surgical method. Due to the risk of persistent tumour cells in the frozen ovarian tissue, OTC/OTT is inadvisable in ovarian cancer and borderline ovarian tumours. Particular caution should be taken in case of leukaemia, certain lymphomas, tumours of the central nervous system (neuroblastoma, medulloblastoma) and in patients carrying the BRCA mutation. In the latter group, it is preferable to perform oocyte or embryo cryopreservation, and if OTC/OTT is performed, the ovarian tissue should be removed after the completion of reproductive plans. In all cases of OTC/OTT, a histopathological examination for the presence of persistent cancer cells in the transplanted tissue is recommended. In order to minimise the risk of bacterial infections, the strict microbiological control of each stage of the cryopreservation, thawing and transport of the tissue is recommended. The use of prophylactic antibiotic therapy prior to surgery is also advised [83]. To date, there are no data that indicate that OTC/OTT contributes to an increased risk of birth defects and chromosomal aberrations in the offspring.

### 4.3. Oocyte In Vitro Maturation IVM

In patients in whom gonadotoxic treatment cannot be deferred until controlled ovarian hyperstimulation is completed or in whom ovarian stimulation is contraindicated, and in prepubertal girls, a novel oocyte in vitro maturation (IVM) procedure may be considered. IVM involves the collection of germinal vesicle (GV)-stage cumulus–oocyte complexes (COCs) from small antral follicles, which are then matured in vitro until they reach the metaphase of the second meiotic division. The procedure is performed irrespective of the menstrual cycle phase and with no ovarian hyperstimulation process. The obtained oocytes undergo maturation and then cryopreservation either immediately after maturation or after fertilisation. Potentially, oocytes could undergo freezing prior to the maturation process, at the germinal vesicle stage, but the available data demonstrate a greater capacity to mature when IVM is performed prior to vitrification [83]. The live birth rate after IVM is around 38% [94]. However, according to Chian et al., it differs depending on the source of immature oocytes, the number of obtained oocytes and the type of IVM protocol [95].

### 4.4. Hormonal Ovarian Suppression

There is much controversy surrounding the use of GnRH analogues as a method of fertility preservation. However, some of the randomised trials that have been conducted prove the efficacy of this non-invasive, simple-to-use and relatively inexpensive method for reducing the risk of POI symptoms after chemotherapy [96,97]. In most studies, the evaluated features after oncological therapy included the recurrence of menstrual bleedings and ovulation, which does not always go hand in hand with the preservation of the ovarian reserve and achievement of pregnancy. The only study that used the number of achieved pregnancies as a secondary endpoint was the POEMS study [98]. It was proven in the study that the pregnancy rate of women who received chemotherapy with goserelin was 23.1%, while for the group treated with chemotherapy alone, it was only 12.2% [90].

It is difficult to fully explain the protective effect of GnRH on the ovaries, but the following mechanisms of action are considered: (1) the prevention of the gonadotropin level rise resulting in the increased recruitment and maturation of ovarian follicles, (2) the direct effect on the ovaries via the GnRH receptors present on the ovaries with an inhibiting effect on cell apoptosis, (3) pituitary desensitisation leading to hypoestrogenism and in turn decreased uterine–ovarian perfusion with the reduced exposure of the ovarian tissue to chemotherapeutic agents, (4) the increased synthesis of ovarian protective substances such as sphingosine-1-phosphate (S1P) and its analogues, potentially inhibiting chemotherapy-induced oocyte apoptosis and (5) the hypothetical protection of undifferentiated ovarian germinal stem cells (GSCs), which potentially could renew the pool of primordial follicles [99]. Side effects of GnRH analogue therapy include the occurrence of hypoestrogenism symptoms, i.e., hot flashes, increased bone turnover, vaginal dryness, decreased libido and emotional lability, and may overlap with the side effects of anticancer therapies. According to the ESHRE guidelines, the initiation of GnRH analogue therapy should be an option for women undergoing chemotherapy for breast cancer, possibly ovarian cancer and lymphomas, in order to protect ovarian function. With regard to fertility preservation, it should give way to other methods and be used only when these are not possible.

### 4.5. Ovarian Transposition—Oophoropexy

Oophoropexy is a method involving the surgical transfer of the ovaries to a location that is not exposed to radiation. Using open, laparoscopic or robotic surgical methods, there is a possibility to perform either medial or preferably lateral ovarian transposition. The former relocates the ovaries behind the uterus. Meanwhile, the most common sites for lateral transposition are the paracolic gutters just above the pelvic rim [100]. The success rate of this procedure in terms of ovarian function return evaluated by post-treatment gonadotropin levels is determined to be 88.6% [101]. The method should not be considered in women awaiting treatment with chemotherapy or in those with low ovarian reserve and possible ovarian metastases [83].

### 4.6. Experimental Methods

**Figure 2:**
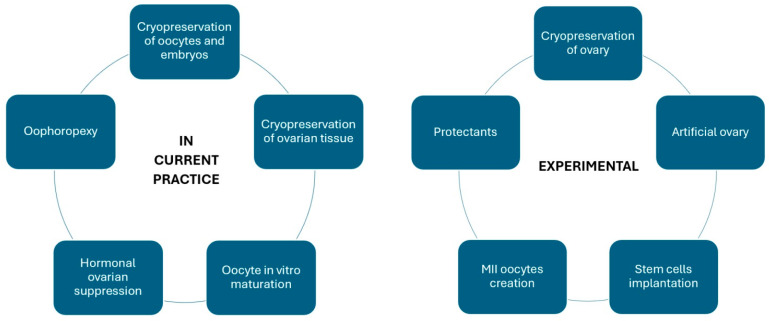
Currently used and experimental oncofertility methods.

The retransplantation of the entire ovary after cryopreservation remains in the realm of research. For the time being, the freezing of the entire organ and its revascularisation are challenging, as is the potential risk of introducing cancer cells into the body [83]. Another method being investigated is the creation of an artificial ovary for transplantation. Preantral follicles isolated from biopsied ovarian tissue could be placed on a special 3D scaffold to protect their structure during transplantation into the body. After the degradation of the scaffold, their growth, migration, proliferation and vascularisation would be possible. The search for the most suitable biomaterial for scaffolds is currently underway [102,103]. Another method based on transplantation is the implantation of different types of cells, including mesenchymal stem cells, undifferentiated ovarian cortex GSCs or induced pluripotent stem cells (iPSCs), into the gonadotoxic-treated ovaries and the activation of primordial follicles within them [83]. Research is also underway to produce oocytes at the metaphase of the second meiotic division from different types of cells derived from the ovarian cortex or from outside the ovary [83]. In addition, various substances are being investigated to protect the ovaries from the toxic effects of chemotherapy, such as sphingosine-1-phosphate (S1P), dexrazoxane, bortezomib and tyrosine kinase inhibitors [45]. The range of currently used and experimental methods is summarised in Figure 2.

## 5. European Perspective

Available guidelines from European countries emphasise the importance of accurately informing patients about the concept and availability of fertility preservation methods before the initiation of gonadotoxic treatment, preferably already at the visit when the patient is informed about the cancer diagnosis. The information should be provided verbally and in writing and recorded in the medical history. Undertaking fertility-preserving procedures should not affect the success of the oncology treatment; hence, in case of any doubt, anticancer therapy takes priority over fertility-preserving treatment. During the various stages of the oncofertility process, there is an emphasis on collaboration between physicians in multi-specialist teams consisting of oncologists, oncologic surgeons, radiotherapists, gynaecologists and reproductive medicine specialists. A patient awaiting gonadotoxic treatment should be promptly reviewed by reproductive specialists. It is important to assign a coordinating role to a member of the medical team to mediate between the patient and the medical staff to facilitate the patient’s journey through the oncofertility process. It is advisable to provide patients with access to psychological support and counselling. European recommendations are created by the European Society for Human Reproduction and Embryology (ESHRE) based on an analysis of the data from EU countries. The preferred methods for preserving fertility in women are the cryopreservation of embryos and oocytes. According to the update of the guidelines—ESHRE 2020—ovarian tissue cryopreservation (OTC) and its subsequent retransplantation (OTT) is no longer considered an experimental method. It is treated as a therapeutic method and recommended primarily in prepubertal patients and in whom ovarian stimulation prior to the IVF is not advisable. Oncofertility Poland is an initiative founded in Poland in 2015 as a section of the Polish Society of Oncological Gynaecology. It brings together 12 national centres specialised in fertility preservation. Currently, oncofertility procedures are not refunded in Poland.

## 6. Mental Health

In addition to having a detrimental impact on physical health, including the fertility and endocrine disorders described, cancer treatment also leads to psychological consequences. It has been shown that oncology patients undergoing fertility preservation procedures are at higher risk of stress, anxiety and depression compared to infertile but non-oncology patients [104,105]. For this reason, it is recommended that patients should be offered psychological support and counselling extending across all stages of oncological treatment starting from diagnosis to various stages of fertility restoration. The available literature emphasises the relevance of creating specific recommendations which would define the entity responsible for psychological counselling, the scope of counselling and appropriate tailoring of the counselling so that it meets the needs of individual patients [105].

## 7. Conclusions

Regardless of the extraordinary progress in the field of oncofertility in the last few decades, further development and attempts to increase its effectiveness and availability are still required as the survival rates of young women with cancer are projected to increase by about 20% in the next 10 years [8]. Oncological treatments lead to fertility impairment causing primary ovarian insufficiency and derangements to all tests assessing the reproductive potential including AMH, AFC, inhibin B, FSH and E2. Among chemotherapeutics, the highest risk of fertility loss is posed by alkylating agents and doxorubicin. The risk of follicle damage caused by radiotherapy depends on the dose, the type of field irradiated, the fractionation scheme and the phase of follicle development at the time of therapy. Surgical treatment may also reduce the pool of ovarian follicles, with adnexal procedures being the most risky. The first-line fertility preservation method remains the cryopreservation of oocytes or embryos. Apart from providing the best cancer treatment and fertility preservation and restoration methods, it is recommended that patients should also be offered psychological support and counselling extending across all stages of their treatment.

## Figures and Tables

**Table 1:** Reproductive potential assessment methods.

Method	Type of Measurement	Timing of Measurement in the Menstrual Cycle	Radiotherapy Impact [40]	Chemotherapy Impact
AMH	Laboratory	Independent	Decreased	Decreased [30]
AFC	Ultrasonography	Day 2–4	Decreased	Decreased [37]
Inhibin B	Laboratory	Day 2	Decreased	Decreased [39]
FSH, E2	Laboratory	Day 2–4	Increased, Decreased	Increased, Decreased [39]

**Table 2:** Chemotherapeutic drugs, their mechanisms of action and effect on ovarian follicles.

Class of Chemotherapeutic Drug	Mechanism of Action	Type of Follicles Affected	Effect on Follicles
Alkylating agents	Damaging the DNA strand structure by alkylating the nitrogen atoms of the nucleotide bases leading to forming cross-links in the DNA, Blocking enzymes mediating DNA repair processes	Primordial, Growing	Increased activation, Atresia through damaging oocytes
Antitumour antibiotics ^1^	Disruption of the DNA structure by intercalation between base pairs leading to changes in helix torsion angle, Inhibition of topoisomerase II activity, Disruption of mitochondrial function, Exacerbation of cellular oxidative stress	Primordial, Growing	Increased activation, Quality deterioration, Atresia through damaging granulosa cells
Antimetabolites	Competitive blockage of the enzymatic reactions responsible for cell growth	Growing	Growth inhibition, Atresia
Taxanes	Inhibition of mitotic spindle function, Inducing cells’ return to the cell cycle G phase	Growing	Atresia ^2^
Vinca alkaloids	Inhibition of mitotic spindle function	Growing	Atresia ^2^

^1^ By the example of doxorubicin, which belongs to the group of anthracyclines. ^2^ The results have been obtained only in animal models.

## Data Availability

No new data were created or analyzed in this study. Data sharing is not applicable to this article.

## Abbreviations

AYA—adolescents and young adults, POI/POF—primary ovarian insufficiency, IVM—in vitro maturation, AMH—anti-Mullerian hormone, FSH—follicle-stimulating hormone, E2—oestradiol, AFC—antral follicle count, TGFβ—transforming growth factor β, OHSS—ovarian hyperstimulation syndrome, TBI—total body irradiation, OTC—ovarian tissue cryopreservation, OTT—ovarian tissue transplantation, COH—controlled ovarian hyperstimulation.

## References

[1] Sung H., Ferlay J., Siegel R.L., Laversanne M., Soerjomataram I., Jemal A., Bray F. (2021). Global Cancer Statistics 2020: GLOBOCAN Estimates of Incidence and Mortality Worldwide for 36 Cancers in 185 Countries. CA Cancer J Clin..

[2] WHO International Agency for Research on Cancer. https://www.iarc.who.int.

[3] Miller K.D., Nogueira L., Devasia T., Mariotto A.B., Yabroff K.R., Jemal A., Kramer J., Siegel R.L. (2022). Cancer treatment and survivorship statistics; 2022. CA Cancer J Clin..

[4] American Cancer Society. https://www.cancer.org/cancer/types/cancer-in-children/key-statistics.html.

[5] National Cancer Institute Cancer Statistics. https://seer.cancer.gov/statfacts/html/aya.html.

[6] Trama A., Stark D., Bozovic-Spasojevic I., Gaspar N., Peccatori F., Toss A., Bernasconi A., Quarello P., Scheinemann K., Jezdic S. (2023). Cancer burden in adolescents and young adults in Europe. ESMO Open.

[7] WHO Report “Childhood Cancer Inequalities in the WHO European Region”. https://www.who.int/europe/news/item/15-02-2022-new-who-report-highlights-scale-of-childhood-cancer-inequalities-in-the-european-region.

[8] National Cancer Institute. https://cancercontrol.cancer.gov/ocs/statistics#stats.

[9] Ataman L.M., Rodrigues J.K., Marinho R.M., Caetano J.P., Chehin M.B., Alves da Motta E.L., Serafini P., Suzuki N., Furui T., Takae S. (2016). Creating a Global Community of Practice for Oncofertility. J. Glob. Oncol..

[10] Woodruff T.K., Ataman-Millhouse L., Acharya K.S., Almeida-Santos T., Anazodo A., Anderson R.A., Appiah L., Bader J., Becktell K., Brannigan R.E. (2021). A View from the past into our collective future: The oncofertility consortium vision statement. J. Assist. Reprod. Genet..

[11] De Vos M., Smitz J., Woodruff T.K. (2014). Fertility preservation in women with cancer. Lancet.

[12] Carson S.A., Kallen A.N. (2021). Diagnosis and Management of Infertility: A Review. JAMA.

[13] Bala R., Singh V., Rajender S., Singh K. (2021). Environment, Lifestyle, and Female Infertility. Reprod. Sci..

[14] Gelbaya T.A., Potdar N., Jeve Y.B., Nardo L.G. (2014). Definition and epidemiology of unexplained infertility. Obstet. Gynecol. Surv..

[15] Webber L., Davies M., Anderson R., Bartlett J., Braat D., Cartwright B., Cifkova R., de Muinck Keizer-Schrama S., Hogervorst E., European Society for Human Reproduction and Embryology (ESHRE) Guideline Group on POI (2016). ESHRE Guideline: Management of women with premature ovarian insufficiency. Hum. Reprod..

[16] Ishizuka B. (2021). Current Understanding of the Etiology, Symptomatology, and Treatment Options in Premature Ovarian Insufficiency (POI). Front. Endocrinol..

[17] Chen M., Jiang H., Zhang C. (2023). Selected Genetic Factors Associated with Primary Ovarian Insufficiency. Int. J. Mol. Sci..

[18] Takahashi A., Yousif A., Hong L., Chefetz I. (2021). Premature ovarian insufficiency: Pathogenesis and therapeutic potential of mesenchymal stem cell. J. Mol. Med..

[19] Szeliga A., Calik-Ksepka A., Maciejewska-Jeske M., Grymowicz M., Smolarczyk K., Kostrzak A., Smolarczyk R., Rudnicka E., Meczekalski B. (2021). Autoimmune Diseases in Patients with Premature Ovarian Insufficiency-Our Current State of Knowledge. Int. J. Mol. Sci..

[20] McClam M., Xiao S. (2022). Preserving Oocytes in Oncofertility. Biol. Reprod..

[21] Tuppi M., Kehrloesser S., Coutandin D.W., Rossi V., Luh L.M., Strubel A., Hötte K., Hoffmeister M., Schäfer B., De Oliveira T. (2018). Oocyte DNA damage quality control requires consecutive interplay of CHK2 and CK1 to activate p63. Nat. Struct. Mol. Biol..

[22] Cedars M.I. (2022). Evaluation of Female Fertility-AMH and Ovarian Reserve Testing. J. Clin. Endocrinol. Metab..

[23] Park S.U., Walsh L., Berkowitz K.M. (2021). Mechanisms of ovarian aging. Reproduction.

[24] Zhu Q., Li Y., Ma J., Ma H., Liang X. (2023). Potential factors result in diminished ovarian reserve: A comprehensive review. J. Ovarian Res..

[25] Josso N., Picard J.Y. (2022). Genetics of anti-Müllerian hormone and its signaling pathway. Best. Pract. Res. Clin. Endocrinol. Metab..

[26] di Clemente N., Racine C., Pierre A., Taieb J. (2021). Anti-Müllerian Hormone in Female Reproduction. Endocr. Rev..

[27] Yildiz S., Bildik G., Benlioglu C., Turan V., Dilege E., Ozel M., Kim S., Oktem O. (2023). Breast cancer treatment and ovarian function. Reprod. Biomed. Online.

[28] Moolhuijsen L.M.E., Visser J.A. (2020). Anti-Müllerian Hormone and Ovarian Reserve: Update on Assessing Ovarian Function. J. Clin. Endocrinol. Metab..

[29] Anderson R.A., Cameron D.A. (2011). Pretreatment serum anti-müllerian hormone predicts long-term ovarian function and bone mass after chemotherapy for early breast cancer. J. Clin. Endocrinol. Metab..

[30] Brougham M.F., Crofton P.M., Johnson E.J., Evans N., Anderson R.A., Wallace W.H. (2012). Anti-Müllerian hormone is a marker of gonadotoxicity in pre- and postpubertal girls treated for cancer: A prospective study. J. Clin. Endocrinol. Metab..

[31] Henry N.L., Xia R., Schott A.F., McConnell D., Banerjee M., Hayes D.F. (2014). Prediction of postchemotherapy ovarian function using markers of ovarian reserve. Oncologist.

[32] Dillon K.E., Sammel M.D., Prewitt M., Ginsberg J.P., Walker D., Mersereau J.E., Gosiengfiao Y., Gracia C.R. (2013). Pretreatment antimüllerian hormone levels determine rate of posttherapy ovarian reserve recovery: Acute changes in ovarian reserve during and after chemotherapy. Fertil. Steril..

[33] Loubersac S., Dezellus A., Lefebvre T., Reignier A., Barriere P., Masson D., Freour T., RESOVA Investigators Group (2020). Evolution of serum Anti-Müllerian Hormone (AMH) level in young women treated with chemotherapy for breast cancer according to basal AMH level. Eur. J. Obstet. Gynecol. Reprod. Biol..

[34] Berjeb K.K., Debbabi L., Braham M., Zemni Z., Chtourou S., Hannachi H., Hamdoun M., Ayadi M., Kacem K., Zhioua F. (2021). Evaluation of ovarian reserve before and after chemotherapy. J. Gynecol. Obstet. Hum. Reprod..

[35] Li H.W.R., Robertson D.M., Burns C., Ledger W.L. (2021). Challenges in Measuring AMH in the Clinical Setting. Front. Endocrinol..

[36] Li H.W.R., Ko J.K.Y., Lee V.C.Y., Yung S.S.F., Lau E.Y.L., Yeung W.S.B., Ho P.C., Ng E.H.Y. (2020). Comparison of antral follicle count and serum anti Müllerian hormone level for determination of gonadotropin dosing in in-vitro fertilization: Randomized trial. Ultrasound Obstet. Gynecol..

[37] Sinha N., Letourneau J.M., Wald K., Xiong P., Imbar T., Li B., Harris E., Mok-Lin E., Cedars M.I., Rosen M.P. (2018). Antral follicle count recovery in women with menses after treatment with and without gonadotropin-releasing hormone agonist use during chemotherapy for breast cancer. J. Assist. Reprod. Genet..

[38] Practice Committee of the American Society for Reproductive Medicine (2020). Testing and interpreting measures of ovarian reserve: A committee opinion. Fertil. Steril..

[39] Su H.I., Sammel M.D., Green J., Velders L., Stankiewicz C., Matro J., Freeman E.W., Gracia C.R., DeMichele A. (2010). Antimullerian hormone and inhibin B are hormone measures of ovarian function in late reproductive-aged breast cancer survivors. Cancer.

[40] Gracia C.R., Sammel M.D., Freeman E., Prewitt M., Carlson C., Ray A., Vance A., Ginsberg J.P. (2012). Impact of cancer therapies on ovarian reserve. Fertil. Steril..

[41] Di Tucci C., Galati G., Mattei G., Chinè A., Fracassi A., Muzii L. (2022). Fertility after Cancer: Risks and Successes. Cancers.

[42] Bedoschi G., Navarro P.A., Oktay K. (2016). Chemotherapy-induced damage to ovary: Mechanisms and clinical impact. Future Oncol..

[43] Korbut R. (2012). Farmakologia.

[44] Yuksel A., Bildik G., Senbabaoglu F., Akin N., Arvas M., Unal F., Kilic Y., Karanfil I., Eryılmaz B., Yilmaz P. (2015). The magnitude of gonadotoxicity of chemotherapy drugs on ovarian follicles and granulosa cells varies depending upon the category of the drugs and the type of granulosa cells. Hum. Reprod..

[45] Spears N., Lopes F., Stefansdottir A., Rossi V., De Felici M., Anderson R.A., Klinger F.G. (2019). Ovarian damage from chemotherapy and current approaches to its protection. Hum. Reprod. Update.

[46] Kalich-Philosoph L., Roness H., Carmely A., Fishel-Bartal M., Ligumsky H., Paglin S., Wolf I., Kanety H., Sredni B., Meirow D. (2013). Cyclophosphamide triggers follicle activation and “burnout”, AS101 prevents follicle loss and preserves fertility. Sci. Transl. Med..

[47] Nguyen Q.N., Zerafa N., Liew S.H., Findlay J.K., Hickey M., Hutt K.J. (2019). Cisplatin- and cyclophosphamide-induced primordial follicle depletion is caused by direct damage to oocytes. Mol. Hum. Reprod..

[48] Nguyen Q.N., Zerafa N., Liew S.H., Morgan F.H., Strasser A., Scott C.L., Findlay J.K., Hickey M., Hutt K.J. (2018). Loss of PUMA protects the ovarian reserve during DNA-damaging chemotherapy and preserves fertility. Cell Death Dis..

[49] Kciuk M., Gielecińska A., Mujwar S., Kołat D., Kałuzińska-Kołat Ż., Celik I., Kontek R. (2023). Doxorubicin-An Agent with Multiple Mechanisms of Anticancer Activity. Cells.

[50] Mattioli R., Ilari A., Colotti B., Mosca L., Fazi F., Colotti G. (2023). Doxorubicin and other anthracyclines in cancers: Activity, chemoresistance and its overcoming. Mol. Asp. Med..

[51] Lopes F., Liu J., Morgan S., Matthews R., Nevin L., Anderson R.A., Spears N. (2020). Single and combined effects of cisplatin and doxorubicin on the human and mouse ovary in vitro. Reproduction.

[52] Morgan S., Lopes F., Gourley C., Anderson R.A., Spears N. (2013). Cisplatin and doxorubicin induce distinct mechanisms of ovarian follicle loss, imatinib provides selective protection only against cisplatin. PLoS ONE.

[53] Tarasiewicz M., Martynowicz I., Knapp P., Sieczyński P. (2019). “Oncofertility” procedures in children and adolescents. Pediatr. Endocrinol. Diabetes Metab..

[54] Ben-Aharon I., Bar-Joseph H., Tzarfaty G., Kuchinsky L., Rizel S., Stemmer S.M., Shalgi R. (2010). Doxorubicin-induced ovarian toxicity. Reprod. Biol. Endocrinol..

[55] Almeida J.Z., Lima L.F., Vieira L.A., Maside C., Ferreira A.C.A., Araújo V.R., Duarte A.B.G., Raposo R.S., Báo S.N., Campello C.C. (2021). 5-Fluorouracil disrupts ovarian preantral follicles in young C57BL6J mice. Cancer Chemother. Pharmacol..

[56] Wu C., Wu T., Chen D., Wei S., Tang W., Xue L., Xiong J., Huang Y., Guo Y., Chen Y. (2022). The effects and mechanism of taxanes on chemotherapy-associated ovarian damage: A review of current evidence. Front. Endocrinol..

[57] Furlanetto J., Marmé F., Seiler S., Thode C., Untch M., Schmatloch S., Schneeweiss A., Bassy M., Fasching P.A., Strik D. (2021). Chemotherapy-induced ovarian failure in young women with early breast cancer: Prospective analysis of four randomised neoadjuvant/adjuvant breast cancer trials. Eur. J. Cancer.

[58] Iwamoto T., Hara F., Uemura Y., Mukai H., Watanabe T., Ohashi Y. (2020). NSAS-BC02 substudy of chemotherapy-induced amenorrhea (CIA) in premenopausal patients who received either taxane alone or doxorubicin(A) cyclophosphamide(C) followed by taxane as postoperative chemotherapy. Breast Cancer Res. Treat..

[59] Lambertini M., Olympios N., Lequesne J., Calbrix C., Fontanilles M., Loeb A., Leheurteur M., Demeestere I., Di Fiore F., Perdrix A. (2019). Impact of Taxanes, Endocrine Therapy, and Deleterious Germline *BRCA* Mutations on Anti-müllerian Hormone Levels in Early Breast Cancer Patients Treated with Anthracycline- and Cyclophosphamide-Based Chemotherapy. Front. Oncol..

[60] Winship A.L., Carpenter M., Griffiths M., Hutt K.J. (2019). Vincristine Chemotherapy Induces Atresia of Growing Ovarian Follicles in Mice. Toxicol. Sci..

[61] Adriaens I., Smitz J., Jacquet P. (2009). The current knowledge on radiosensitivity of ovarian follicle development stages. Hum. Reprod. Update.

[62] Dolmans M.M., von Wolff M., Poirot C., Diaz-Garcia C., Cacciottola L., Boissel N., Liebenthron J., Pellicer A., Donnez J., Andersen C.Y. (2021). Transplantation of cryopreserved ovarian tissue in a series of 285 women: A review of five leading European centers. Fertil. Steril..

[63] Wallace W.H., Thomson A.B., Kelsey T.W. (2003). The radiosensitivity of the human oocyte. Hum. Reprod..

[64] Wallace W.H., Thomson A.B., Saran F., Kelsey T.W. (2005). Predicting age of ovarian failure after radiation to a field that includes the ovaries. Int. J. Radiat. Oncol. Biol. Phys..

[65] Jayasinghe Y.L., Wallace W.H.B., Anderson R.A. (2018). Ovarian function, fertility and reproductive lifespan in cancer patients. Expert Rev. Endocrinol. Metab..

[66] Kim S., Kim S.W., Han S.J., Lee S., Park H.T., Song J.Y., Kim T. (2021). Molecular Mechanism and Prevention Strategy of Chemotherapy- and Radiotherapy-Induced Ovarian Damage. Int. J. Mol. Sci..

[67] Griffiths M.J., Winship A.L., Hutt K.J. (2020). Do cancer therapies damage the uterus and compromise fertility?. Hum. Reprod. Update.

[68] van de Loo L.E.X.M., van den Berg M.H., Overbeek A., van Dijk M., Damen L., Lambalk C.B., Ronckers C.M., van den Heuvel-Eibrink M.M., Kremer L.C.M., van der Pal H.J. (2019). Uterine function, pregnancy complications, and pregnancy outcomes among female childhood cancer survivors. Fertil. Steril..

[69] Dolmans M.M., Hossay C., Nguyen T.Y.T., Poirot C. (2021). Fertility Preservation: How to Preserve Ovarian Function in Children, Adolescents and Adults. J. Clin. Med..

[70] Teh W.T., Stern C., Chander S., Hickey M. (2014). The impact of uterine radiation on subsequent fertility and pregnancy outcomes. Biomed. Res. Int..

[71] Littley M.D., Shalet S.M., Beardwell C.G., Robinson E.L., Sutton M.L. (1989). Radiation-induced hypopituitarism is dose-dependent. Clin Endocrinol..

[72] Kufel-Grabowska J., Łukaszuk K., Błażek M., Jagiełło-Gruszfeld A., Horbaczewska A., Irga-Jaworska N., Jach R., Jędrzejczak P., Kopeć I., Krawczuk-Rybak M. (2023). Fertility preservation during oncological treatment. Oncol. Clin. Pract..

[73] Cibula D., Raspollini M.R., Planchamp F., Centeno C., Chargari C., Felix A., Fischerová D., Jahnn-Kuch D., Joly F., Kohler C. (2023). ESGO/ESTRO/ESP Guidelines for the management of patients with cervical cancer—Update 2023. Int. J. Gynecol. Cancer.

[74] Trojano G., Olivieri C., Tinelli R., Damiani G.R., Pellegrino A., Cicinelli E. (2019). Conservative treatment in early stage endometrial cancer: A review. Acta Biomed..

[75] Gallo A., Catena U., Saccone G., Di Spiezio Sardo A. (2021). Conservative Surgery in Endometrial Cancer. J. Clin. Med..

[76] Lucchini S.M., Esteban A., Nigra M.A., Palacios A.T., Alzate-Granados J.P., Borla H.F. (2021). Updates on conservative management of endometrial cancer in patients younger than 45 years. Gynecol. Oncol..

[77] Rodolakis A., Scambia G., Planchamp F., Acien M., Di Spiezio Sardo A., Farrugia M., Grynberg M., Pakiz M., Pavlakis K., Vermeulen N. (2023). ESGO/ESHRE/ESGE Guidelines for the fertility-sparing treatment of patients with endometrial carcinoma. Hum. Reprod. Open.

[78] Floyd J.L., Campbell S., Rauh-Hain J.A., Woodard T. (2021). Fertility preservation in women with early-stage gynecologic cancer: Optimizing oncologic and reproductive outcomes. Int. J. Gynecol. Cancer.

[79] Kim S.Y., Lee J.R. (2016). Fertility preservation option in young women with ovarian cancer. Future Oncol..

[80] Colombo N., Sessa C., du Bois A., Ledermann J., McCluggage W.G., McNeish I., Morice P., Pignata S., Ray-Coquard I., Vergote I. (2019). ESMO-ESGO consensus conference recommendations on ovarian cancer: Pathology and molecular biology, early and advanced stages, borderline tumours and recurrent disease†. Ann. Oncol..

[81] Santos M.L., Pais A.S., Almeida Santos T. (2021). Fertility preservation in ovarian cancer patients. Gynecol. Endocrinol..

[82] Basta A., Bidziński M., Bieńkiewicz A., Blecharz P., Bodnar L., Jach R., Knapp P., Kojs Z., Kotarski J., Markowska J. (2015). Recommendation of the Polish Society of Oncological Gynecology on the diagnosis and treatment of epithelial ovarian cancer. Oncol. Clin. Pract..

[83] Anderson R.A., Amant F., Braat D., D’Angelo A., Chuva de Sousa Lopes S.M., Demeestere I., Dwek S., Frith L., Lambertini M., Maslin C. (2020). ESHRE guideline: Female fertility preservation. Hum. Reprod. Open.

[84] Loutradi K.E., Kolibianakis E.M., Venetis C.A., Papanikolaou E.G., Pados G., Bontis I., Tarlatzis B.C. (2008). Cryopreservation of human embryos by vitrification or slow freezing: A systematic review and meta-analysis. Fertil. Steril..

[85] Rezazadeh Valojerdi M., Eftekhari-Yazdi P., Karimian L., Hassani F., Movaghar B. (2009). Vitrification versus slow freezing gives excellent survival, post warming embryo morphology and pregnancy outcomes for human cleaved embryos. J. Assist. Reprod. Genet..

[86] Walker Z., Lanes A., Ginsburg E. (2022). Oocyte cryopreservation review: Outcomes of medical oocyte cryopreservation and planned oocyte cryopreservation. Reprod. Biol. Endocrinol..

[87] Rodriguez-Wallberg K.A., Marklund A., Lundberg F., Wikander I., Milenkovic M., Anastacio A., Sergouniotis F., Wånggren K., Ekengren J., Lind T. (2019). A prospective study of women and girls undergoing fertility preservation due to oncologic and non-oncologic indications in Sweden-Trends in patients’ choices and benefit of the chosen methods after long-term follow up. Acta Obstet. Gynecol. Scand..

[88] Cobo A., García-Velasco J.A., Remohí J., Pellicer A. (2021). Oocyte vitrification for fertility preservation for both medical and nonmedical reasons. Fertil. Steril..

[89] Cobo A., García-Velasco J., Domingo J., Pellicer A., Remohí J. (2018). Elective and Onco-fertility preservation: Factors related to IVF outcomes. Hum. Reprod..

[90] Goldman K.N., Kramer Y., Hodes-Wertz B., Noyes N., McCaffrey C., Grifo J.A. (2015). Long-term cryopreservation of human oocytes does not increase embryonic aneuploidy. Fertil. Steril..

[91] Fraison E., Huberlant S., Labrune E., Cavalieri M., Montagut M., Brugnon F., Courbiere B. (2023). Live birth rate after female fertility preservation for cancer or haematopoietic stem cell transplantation: A systematic review and meta-analysis of the three main techniques; embryo, oocyte and ovarian tissue cryopreservation. Hum. Reprod..

[92] Jach R., Spaczynski R., Kurzawa R., Radwan M., Rzepka J., Swornik M., Pabian W. (2021). Updating the recommendations of the Working Group for the Preservation of Fertility in Oncological and Hematological Patients and Other Patients Treating Gonadier Therapies “ONCOFERTILITY” (GROF) of the Polish Society of Oncological Gynecology regarding cryopreserves and autologous transplant. Ginekol. Pol..

[93] Oktay K.H., Marin L. (2024). Comparison of orthotopic and heterotopic autologous ovarian tissue transplantation outcomes. Fertil. Steril..

[94] Vuong L.N., Le A.H., Ho V.N.A., Pham T.D., Sanchez F., Romero S., De Vos M., Ho T.M., Gilchrist R.B., Smitz J. (2020). Live births after oocyte in vitro maturation with a prematuration step in women with polycystic ovary syndrome. J. Assist. Reprod. Genet..

[95] Chian R.C., Li J.H., Lim J.H., Yoshida H. (2023). IVM of human immature oocytes for infertility treatment and fertility preservation. Reprod. Med. Biol..

[96] Sofiyeva N., Siepmann T., Barlinn K., Seli E., Ata B. (2019). Gonadotropin-Releasing Hormone Analogs for Gonadal Protection During Gonadotoxic Chemotherapy: A Systematic Review and Meta-Analysis. Reprod. Sci..

[97] Leonard R.C.F., Adamson D.J.A., Bertelli G., Mansi J., Yellowlees A., Dunlop J., Thomas G.A., Coleman R.E., Anderson R.A., Anglo Celtic Collaborative Oncology Group and National Cancer Research Institute Trialists (2017). GnRH agonist for protection against ovarian toxicity during chemotherapy for early breast cancer: The Anglo Celtic Group OPTION trial. Ann. Oncol..

[98] Moore H.C.F., Unger J.M., Phillips K.A., Boyle F., Hitre E., Moseley A., Porter D.J., Francis P.A., Goldstein L.J., Gomez H.L. (2019). Final Analysis of the Prevention of Early Menopause Study (POEMS)/SWOG Intergroup S0230. J. Natl. Cancer Inst..

[99] Blumenfeld Z. (2019). Fertility Preservation Using GnRH Agonists: Rationale, Possible Mechanisms, and Explanation of Controversy. Clin. Med. Insights Reprod. Health.

[100] Visvanathan D.K., Cutner A.S., Cassoni A.M., Gaze M., Davies M.C. (2003). A new technique of laparoscopic ovariopexy before irradiation. Fertil. Steril..

[101] Arian S.E., Goodman L., Flyckt R.L., Falcone T. (2017). Ovarian transposition: A surgical option for fertility preservation. Fertil. Steril..

[102] Amorim C.A., Shikanov A. (2016). The artificial ovary: Current status and future perspectives. Future Oncol..

[103] Cho E., Kim Y.Y., Noh K., Ku S.Y. (2019). A new possibility in fertility preservation: The artificial ovary. J. Tissue Eng. Regen. Med..

[104] Lawson A.K., Klock S.C., Pavone M.E., Hirshfeld-Cytron J., Smith K.N., Kazer R.R. (2014). Prospective study of depression and anxiety in female fertility preservation and infertility patients. Fertil. Steril..

[105] Logan S., Anazodo A. (2019). The psychological importance of fertility preservation counseling and support for cancer patients. Acta Obstet. Gynecol. Scand..

